# Fibroblast reticular cells engineer a blastema extracellular network during digit tip regeneration in mice

**DOI:** 10.1002/reg2.75

**Published:** 2017-05-03

**Authors:** Luis Marrero, Jennifer Simkin, Mimi Sammarco, Ken Muneoka

**Affiliations:** ^1^ Department of Cell and Molecular BiologyTulane UniversityNew OrleansLA70118USA; ^2^ Department of MedicineLouisiana State University Health Sciences CenterNew OrleansLA70112USA; ^3^ Department of Veterinary Physiology & PharmacologyTexas A&M UniversityCollege StationTX77843USA

**Keywords:** blastema, digit, regeneration, fibroblast, matrix, ER‐TR7, COL3

## Abstract

The regeneration blastema which forms following amputation of the mouse digit tip is composed of undifferentiated cells bound together by an organized network of fibers. A monoclonal antibody (ER‐TR7) that identifies extracellular matrix (ECM) fibers produced by fibroblast reticular cells during lymphoid organogenesis was used to characterize the ECM of the digit, the blastema, and the regenerate. Digit fibroblast reticular cells produce an ER‐TR7^+^ ECM network associated with different tissues and represent a subset of loose connective tissue fibroblasts. During blastema formation there is an upregulation of matrix production that returns to its pre‐existing level and anatomical pattern in the endpoint regenerate. Co‐localization studies demonstrate a strong spatial correlation between the ER‐TR7 antigen and collagen type III (COL3) in histological sections. ER‐TR7 and COL3 are co‐induced in cultured digit fibroblasts following treatment with tumor necrosis factor alpha and a lymphotoxin beta receptor agonist. These results provide an initial characterization of the ECM during digit regeneration and identify a subpopulation of fibroblasts involved in producing the blastema provisional matrix that is remodeled during the regeneration response.

## 1 INTRODUCTION

The mouse digit tip consists of a diverse group of cells and extracellular components organized into tissue compartments that include the terminal phalangeal bone (P3) with marrow, articular cartilage, tendon, blood vessels, and nerve surrounded by connective tissue (CT), epidermis, and the nail rudiment. The major difference between this structure and other similar mammalian extremities is that the digit tip can regenerate following amputation (Borgens, 1982; Muller et al., 1999; Neufeld, 1985). This phenomenon has been well documented in neonatal and adult mice (Fernando et al., 2011; Han, Yang, Lee, Allan, & Muneoka, 2008) as well as in humans, which makes it clinically relevant (Allan et al., 2006; Muller et al., 1999). Similar to the epimorphic regenerative response that occurs following amputation of a salamander limb, mouse digit tip regeneration involves a sequence of events that include an inflammatory cascade, histolysis of the bone stump, formation of a wound epidermis, blastema growth, and redifferentiation (Fernando et al., 2011; Simkin et al., 2015). The digit tip blastema is a dense mass of proliferating, undifferentiated mesenchymal cells that are derived from multiple tissue types, and many of the regeneration‐competent cell types have been shown to be lineage restricted (Lehoczky, Robert, & Tabin, 2011; Rinkevich, Lindau, Ueno, Longaker, & Weissman, 2011). Digit tip regeneration is an amputation‐level‐specific healing event that diverges from a more typical non‐regenerative wound healing response. For example, amputation at the level of the second phalanx (P2) results in a healing response that also includes an inflammatory cascade, histolysis, and formation of a wound epidermis. However, in contrast with a P3 amputation, this wound fails to form a blastema and develops a fibrotic scar instead (Dawson et al., 2016). Since fibroblast cells derived from this regeneration‐incompetent region are capable of participating in blastema formation (Wu et al., 2013), it seems likely that one difference between regenerative and non‐regenerative responses involves the microenvironment associated with blastema formation.

Many of the cells within the digit blastema appear spindle‐shaped and there is evidence that fibroblasts associated with the CT of the amputated stump participate in blastema formation (Wu et al., 2013). In salamander limb regeneration, there is considerable evidence that CT fibroblasts of the dermis play an important role in blastema formation and patterning during a regenerative response (Bryant, Endo, & Gardiner, 2002; Nacu et al., 2013). Fibroblasts have also been shown to play a key role in the development, function, and repair of mammalian lymphoid organs (e.g., lymph nodes, spleen, and thymus), where a subset of cells called fibroblastic reticular cells (FRCs) form a network of extracellular matrix (ECM) fibers that define B‐cell and T‐cell compartments (Fletcher, Acton, & Knoblich, 2015). Lymph node FRCs also play a role in directing leukocyte migration and are required for antibody generation (Heesters, Myers, & Carroll, 2014; Katakai et al., 2008). Lymph node FRCs and the ECM network they produce can be identified by an antigen recognized by the Erasmus of Rotterdam thymic reticulum, or ER‐TR7, antibody (Van Vliet, Melis, & Van Ewijk, 1984; Van Vliet, Melis, Foidart, & Van Ewijk, 1986). The ER‐TR7 antibody reacts against an unknown epitope that localizes to the membrane or cytosol of FRCs, or both, and the extracellular network of fibers that extends from these FRCs and, as such, has been used to define FRCs (Bajenoff et al., 2006; Balogh, Horvath, & Szakal, 2004; Katakai, Hara, Sugai, Gonda, & Shimizu, 2004b; Link et al., 2011; Nolte et al., 2003).

Current approaches to enhancing the regeneration of human limb structures involve the use of scaffolds that can be seeded with cells ex vivo or become populated with cells after implantation (Quijano, Lynch, Allan, Badylak, & Ahsan, 2016). Scaffolds are ECM structures that can be biological (Badylak, Freytes, & Gilbert, 2009) or synthetic (Wolf, Dearth, Sonnenberg, Loboa, & Badylak, 2015), and can be molded into distinct forms that approximate the target structure, or they can be derived from the decellularization of tissues or an entire organ, such as the heart, lung, or limb (Jank et al., 2015; Ott et al., 2008; Stabler et al., 2015). In some respects, the use of scaffolds in regenerative engineering provides a way to bypass the developmental processes that drive morphogenesis during regeneration. In tissue regeneration and repair, the ECM plays a key role in regulating the injury response. For example, it has been known for some time that in the healing of full‐thickness skin wounds a transient matrix that is high in collagen type III (COL3), called granulation tissue, is produced by inflammatory cells and invading fibroblasts, and is later replaced by a dense fiber network made of collagen type I (COL1) fibers that is the hallmark of scar tissue (Gay, Vijanto, Raekallio, & Penttinen, 1978; Merkel, DiPaolo, Hallock, & Rice, 1988; Whitby & Ferguson, 1991). In bone fracture healing, a chondrogenic callus formed by periosteal cells creates a transient matrix that is later remodeled to form new bone that repairs the damaged bone (Colnot, 2009). In digit tip regeneration, the bone matrix of the stump is degraded by the activity of osteoclasts and this degradation response is linked to the formation of a blastema that mediates the regenerative response (Simkin et al., 2015). Thus, there is a biological basis that supports the clinical potential of scaffold use in regenerative medicine; however, our understanding of matrices that form during regenerative responses remains poor.

In this study, the subset of fibroblastic cells responsible for producing a fraction of the ECM in the unamputated digit tip and the majority of the blastema ECM of the regenerate are characterized by reactivity to the ER‐TR7 antibody and therefore denominated digit FRCs. Various tissues of the digit tip are compartmentalized by an ER‐TR7^+^ component of the ECM produced by digit FRCs which outlines the vasculature, bone, and epidermis. Blastema formation is characterized by an increase in ER‐TR7 immunohistochemistry (IHC) staining which returns to pre‐amputation levels after completion of the regenerative response. A similar increase was not observed in other fibroblast markers analyzed. During blastema formation, ER‐TR7^+^ cells display an enhanced proliferation index compared to ER‐TR7^−^ cells indicating that the blastema microenvironment is specifically mitogenic for digit FRCs. Primary cultured blastema cells maintain expression of the ER‐TR7 antigen whereas cultures of fibroblasts from the digit tip display a low level of reactivity. As has been previously shown for lymphoid FRCs (Katakai et al., 2004a), reactivity to the ER‐TR7 antibody by cultured digit fibroblasts is induced by co‐treatment with tumor necrosis factor alpha (TNFα) and a lymphotoxin T beta receptor (LTβR) agonist. Induction of the ER‐TR7 antigen is associated with an increase in *Col3a1* expression as well as IHC for COL3. Co‐localization analysis indicates a tight association between COL3 and the ER‐TR7 antigen both in vitro and in vivo. These studies provide evidence that digit FRCs react to amputation injury and play a role in producing a network of ECM fibers characteristic of the regenerating digit blastema.

## 2 RESULTS

### 2.1 ER‐TR7 outlines tissue compartments of the neonatal and adult digit anlagen

**Figure 1:**
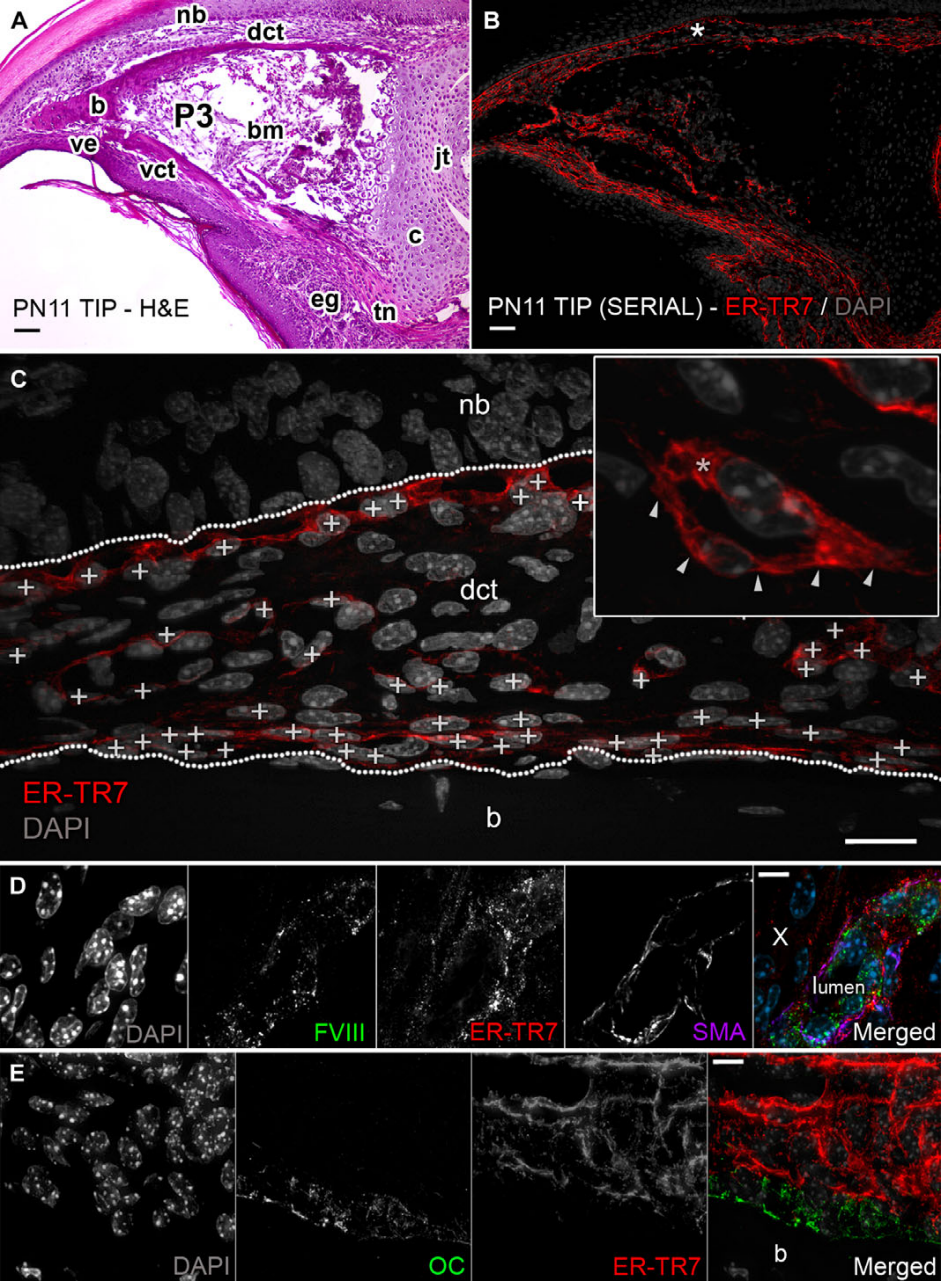
ER‐TR7 outlines tissue compartments of the digit. (A) H&E section of PN11 mouse digit tip shows compartments including nail bed (nb), ventral epithelium (ve), eccrine glands (eg), and a P3 rudiment composed of both cortical bone (b) and a proximal cartilaginous (c) growth plate. P3 encloses bone marrow (bm) and ends at the P3−P2 synovial joint (jt). P3 is connected to the proximal musculature through a tendon (tn) and is surrounded by loose dorsal and ventral CT (dct and vct). (B) Adjacent section from (A) stained against ER‐TR7. (C) Representative area captured at 400× from the dct in (B) (white asterisk). The boundary landmarks of the CT (labeled nb and b) are outlined with white dotted lines. ER‐TR7^+^ FRCs are marked (white + signs on nuclei) and these were discriminated (C, inset) at 1000× magnification by ER‐TR7 expression in membrane extensions (white arrows) or cytosol (white asterisk) of individual cells. Scale bars (A), (B) 50 μm and (C) 25 μm. Serial sections were also co‐immunostained for (D) ER‐TR7, FVIII, and SMA (white × marks negative cells) or (E) ER‐TR7 and osteocalcin OC; scale bars (D)−(E) 10 μm

The P3 of the mouse digit tip originates as a chondrogenic skeletal element at embryonic day 14.5 (E14.5) and ossification initiates at E18.5 (Han et al., 2008). By post‐natal day 11 (PN11), the major specialized compartments of the digit tip including the P3 bone and its marrow cavity, ventral tendon, surrounding CT, epidermis, and synovial joint that articulates with P2 are well defined and remain, aside from growth, unaltered throughout adulthood (Fig. 1A). The digit tip is grossly characterized by a nail organ that surrounds P3 dorsally and laterally and a bulbous ventral pad called the “fat” pad. The nail organ consists of a stratified epithelial layer at its proximal end which extends distally to a single layer of keratinocytes, known as the nail bed, underlying a nail plate. The “fat” pad is mainly composed of continuous epidermis, CT, and eccrine glands. In general, the CT of the digit tip appears as a loose mesenchyme primarily composed of fibroblasts with blood vessels infiltrating throughout the tissue.

FRCs in lymphoid tissue have been identified by reactivity to the ER‐TR7 antibody (Van Vliet et al., 1986) but have not yet been studied in a non‐lymphoid organ. ER‐TR7 IHC on sections of mouse digits was used to determine whether there is a similar population of FRCs in the neonatal and adult digit tips. ER‐TR7 IHC identifies cells and ECM fibers that appear to outline different anatomical compartments of the digit (Fig. 1B). Cells that secrete the ER‐TR7 antigen are identified by cytoplasmic and membrane antigen localization (Fig. 1C), and ER‐TR7 stained ECM fibers can be traced to ER‐TR7^+^ cells but are in contact with both ER‐TR7^+^ and ER‐TR7^−^ cells within the CT of the digit tip.

Digit FRCs extend ER‐TR7^+^ fibers that outline individual components of the digit tip reminiscent of the boundaries they establish between the distinct zones of lymphoid organs. To observe this arrangement, we co‐stained PN11 digits with ER‐TR7 and markers specific to layers of bone and vasculature compartments. The vasculature forms a network within the loose CT surrounding P3, and endothelial cells lining the lumen of these vessels can be identified based on von Willebrand factor (FVIII) IHC. Tightly associated with these endothelial cells are α‐smooth muscle actin (SMA)^+^ mural cells in the intima that function in vascular homeostasis. In addition to these two cell types, we also find cells in the outer adventitia layer that react to the ER‐TR7 antibody. These appear closely associated with but distinct from FVIII^+^ and SMA^+^ cells, and are mostly absent in the surrounding CT where vessels are absent (Fig. 1D; white ×, merged panel). A high number of ER‐TR7^+^ cells form a stratified layer of fibroblasts above osteocalcin (OC)^+^ osteoblasts in the periosteum of P3 (Fig. 1E). Finally, a layer of ER‐TR7^+^ cells delineate the boundary between the papillary layer of the loose CT and the stratum basale of the epidermis, a layer that is identifiable by the arrangement of keratinocytes and their nuclei in hematoxylin and eosin (H&E) preparations or with the nuclear fluorescent counterstain 4′,6‐diamidino‐2‐phenylindole (DAPI) on a fluorescent serial section (Fig. 1A, B). These observations suggest that FRCs are present in the mouse digit as a subpopulation of cells localized to the CT that appear to be housed within the boundaries and basement layers around various types of digit tip structures. Thus, ER‐TR7 staining identifies an arrangement of digit FRCs within the CT of the digit tip that appear analogous to FRCs described during the organogenesis of lymphoid organs (Balogh, Fisi, & Szakal, 2008; Katakai et al., 2004a; Link et al., 2007; Van Vliet et al., 1986).

### 2.2 ER‐TR7 is upregulated during digit tip regeneration

**Figure 2:**
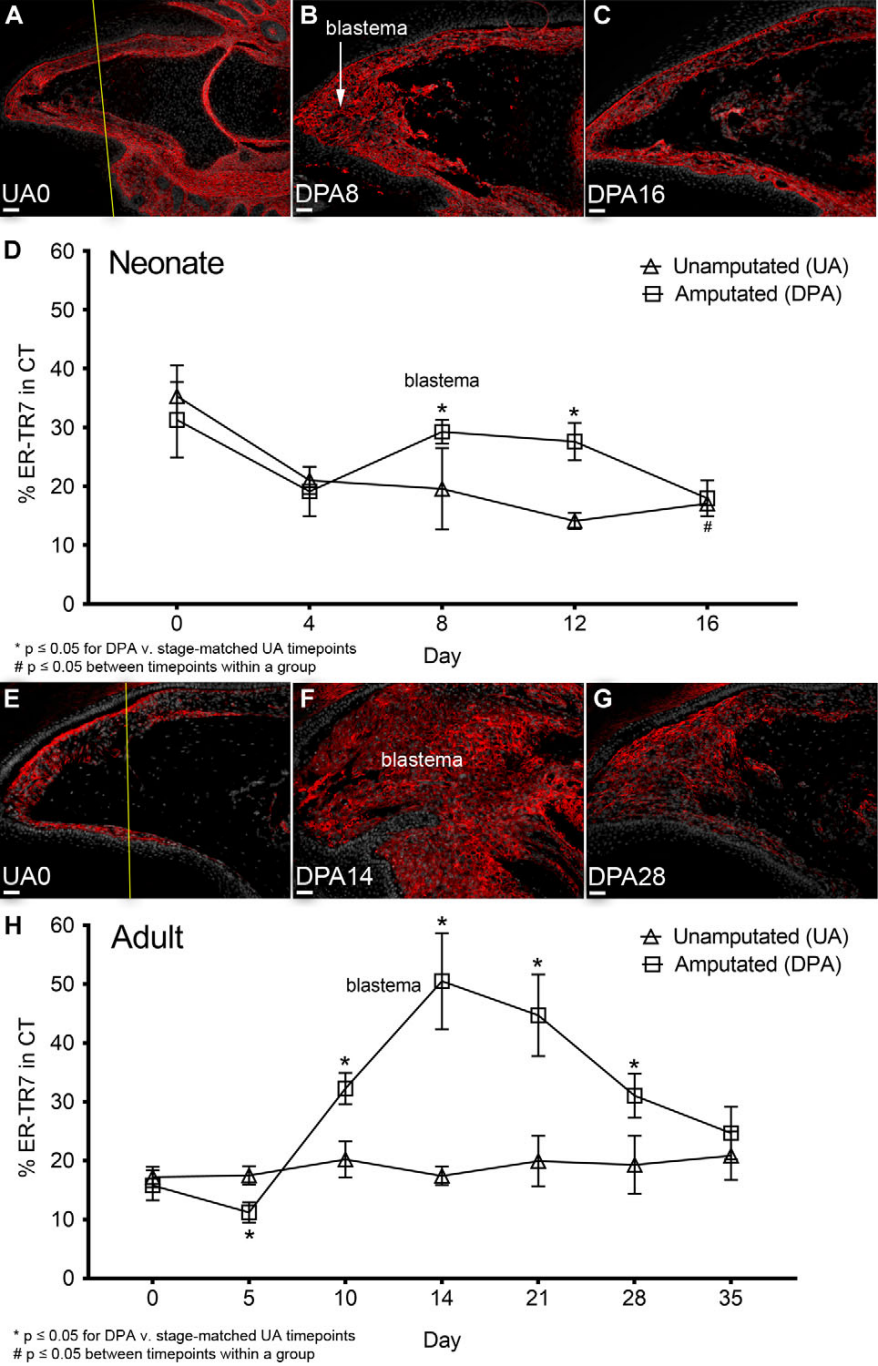
**ER‐TR7 is upregulated during neonatal and adult digit tip blastema formation**. ER‐TR7 (red) expression following digit tip amputation of PN3 neonates and 8W adult mice was detected by indirect immunofluorescence counterstained with DAPI (gray), captured at 100× magnification (scale bar 50 μm), and quantified from groups of UA and DPA digits (*n* = 4 animals per group). Images of select timepoints prior to and following amputation are shown for both (A)−(C) neonates and (E)−(G) adult mouse digit tips. The amputation level for both (A) neonate and (E) adult UA0 digits is marked by a yellow line. ER‐TR7 expression percentages in (D) the neonate and (H) the adult timeline were calculated from the ER‐TR7^+^ area over total CT around each P3 segment and excluding the marrow. Data are presented as mean ± SEM. Scale bars (A)−(D) and (F)−(I) 50 μm

Amputation through the digit tip of both neonates and adult mice is followed by a healing response that forms a blastema of proliferating cells, and these cells redifferentiate to regenerate the amputated structure (Fernando et al., 2011; Han et al., 2008). To determine the anatomical differences and expression profile of ER‐TR7 during the regeneration response, neonates and adult mice were amputated at PN3 or 8 weeks (8W), respectively, and tissues were harvested for ER‐TR7 IHC at various timepoints following amputation (Fig. 2). Subject groups were labeled throughout the study as control unamputated (UA) and days post amputation (DPA) animals followed by the number of days from timepoint 0 when necessary. The difference between the regeneration of neonate and adult digits starting at DPA0 is largely one of timing. Neonate digits are immature and regenerate at a faster rate. Therefore, in the neonate timeline, the endpoint of regeneration is DPA16 with the peak of blastema formation occurring at DPA8 (Han et al., 2008). On the other hand, in the adult timeline, the endpoint of regeneration is DPA35 with the peak of blastema formation occurring around DPA12−14 (Fernando et al., 2011).

ER‐TR7 IHC during regeneration was quantified compared to stage matched UA controls. For neonatal UA digits, ER‐TR7 staining (Fig. 2A) displays a progressive reduction in the relative amount detected in the CT, dropping from approximately 33% of the total area at UA0 to approximately 20% by UA16 (*P* = 0.0005; Fig. 2A and C) and regenerated DPA16 (*P* = 0.01; Fig. 2A and D). The measured level of ER‐TR7 expression in the neonate at UA16 and DPA16 remains stable compared to each other and to U0 of the adult timeline (Fig. 2E and H). Digit regeneration in neonates displays no change in relative ER‐TR7 staining prior to blastema formation, increases during blastema at DPA8 (*P* = 0.04) and early redifferentiation stages at DPA12 (*P* = 0.003) compared to age‐matched UA controls. ER‐TR7 expression then falls to UA16 levels when regeneration is completed by DPA16 (Fig. 2A−D). In adults, there is an initial decline in ER‐TR7 levels during stages DPA0 through DPA5 (*P* = 0.035) and this is followed by a 2‐fold increase in ER‐TR7 staining in the blastema at DPA14 (*P* < 0.0001; Fig. 2F and H) which progressively declines back to the pre‐amputation level (Fig. 2E−H). These data demonstrate that ER‐TR7 expression is transiently enhanced during blastema formation, and that the organization of ER‐TR7^+^ fibers from digit FRCs is modified during the regeneration process.

### 2.3 ER‐TR7^+^ cells are growth responsive during blastema formation

**Figure 3:**
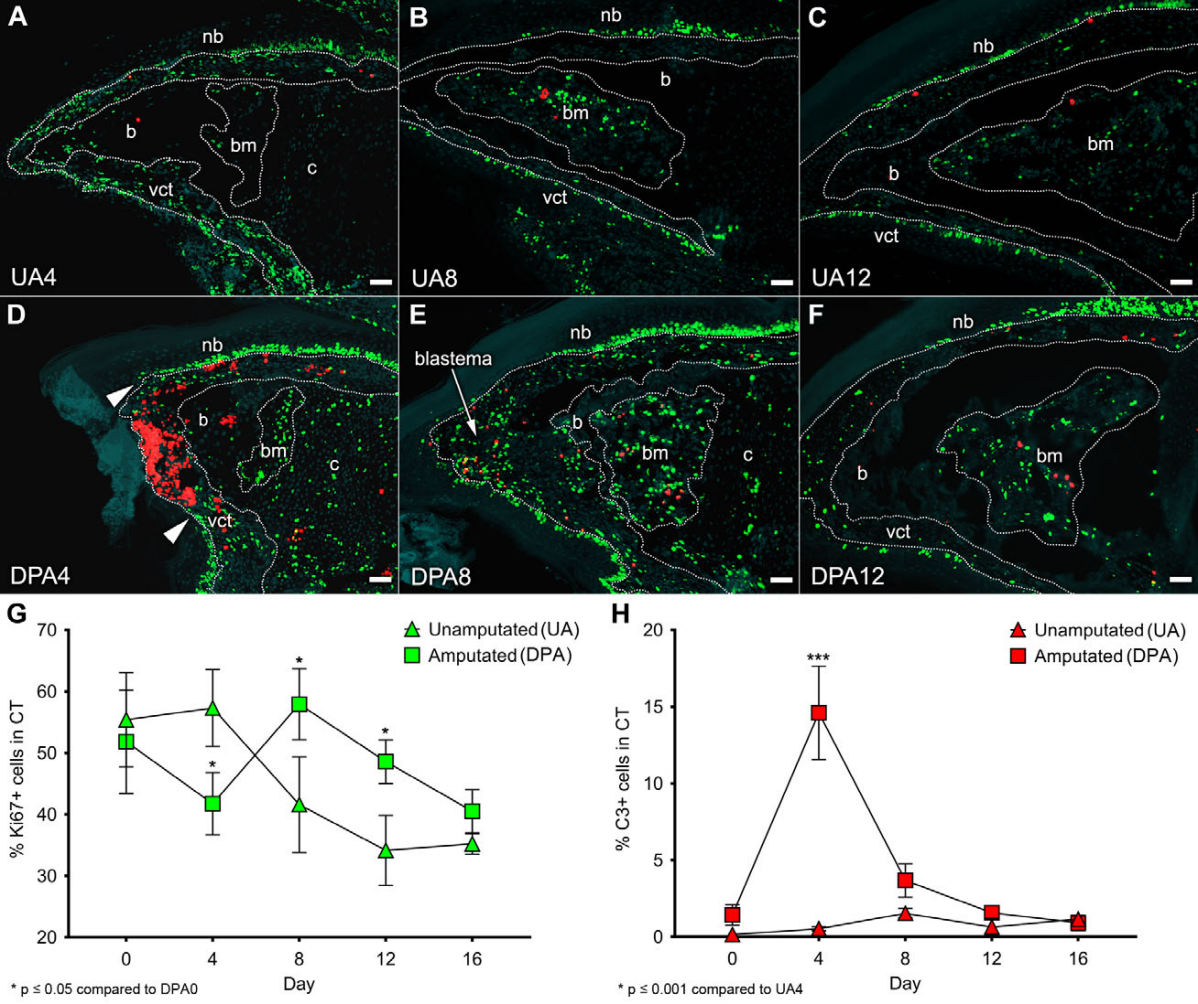
**Proliferation and apoptosis in the regenerating neonatal digit tip**. (A)−(C) Representative UA controls and (D)−(F) age‐matched digits amputated at PN3 were analyzed at DPA0, 4, 8, 12, and 16 for Ki67 (green) and cleaved C3 (red) expression counterstained with DAPI (gray). Nail bed (nb), ventral epidermis (ve), bone (b), chondrocytes (c), and bone marrow (bm) are outlined by white dotted lines; scale bar 50 μm. (G), (H) Ki67^+^ and C3^+^ cells were counted from the CT area outlined between the basal epidermis and the periosteal/perichondrial layers of P3. Data are presented as mean ± SE (*n* = 4 per group and timepoint)

Regulation of the ER‐TR7^+^ microenvironment coincides with periods of tissue degradation, growth, and differentiation after injury. To begin analysis of digit FRCs, a quantitative analysis of overall cell division and apoptosis during neonatal digit tip regeneration was carried out using IHC localization of Ki67 as a proliferation marker and C3 to identify apoptotic cells (Fig. 3). Digits were analyzed at 4‐day intervals from DPA0 to include the early wound healing (DPA4), blastema formation (DPA8), and redifferentiation stages (DPA12 and DPA16). Cell counts were collected from total loose CT of UA digits and both loose and blastema CT of the regenerating digit. Cell proliferation in UA controls displays an age‐related decline (Fig. 3A−C and G), while proliferation associated with regeneration is dynamic (Fig. 3D−G). The early wound healing phase (DPA4) is associated with a decline in proliferation (*P* = 0.021, Fig. 3D and G) and this is followed by a period of enhanced proliferation associated with blastema formation (DPA8; *P* = 0.014, Fig. 3E and G) and early differentiation (DPA12; *P* = 0.004). The proliferation index returns to UA control levels when the regeneration response is completed (DPA16; Fig. 3G). These data show an enhanced proliferative response of cells associated with blastema formation and are similar to previously published studies on regenerating adult digits (Fernando et al., 2011; Wu et al., 2013).

Apoptosis during digit tip regeneration is largely restricted to the early wound healing phase (DPA4; *P* < 0.001, Fig. 3H) and is largely localized to the amputation wound (Fig. 3D, flanked by white arrows). At this timepoint many C3^+^ cells have distinct tri‐lobed nuclei characteristic of granulocytes which link the observed apoptosis to the inflammatory response. There are very few apoptotic cells found within the blastema or during redifferentiation (Fig. 3E, F, and H).

**Figure 4:**
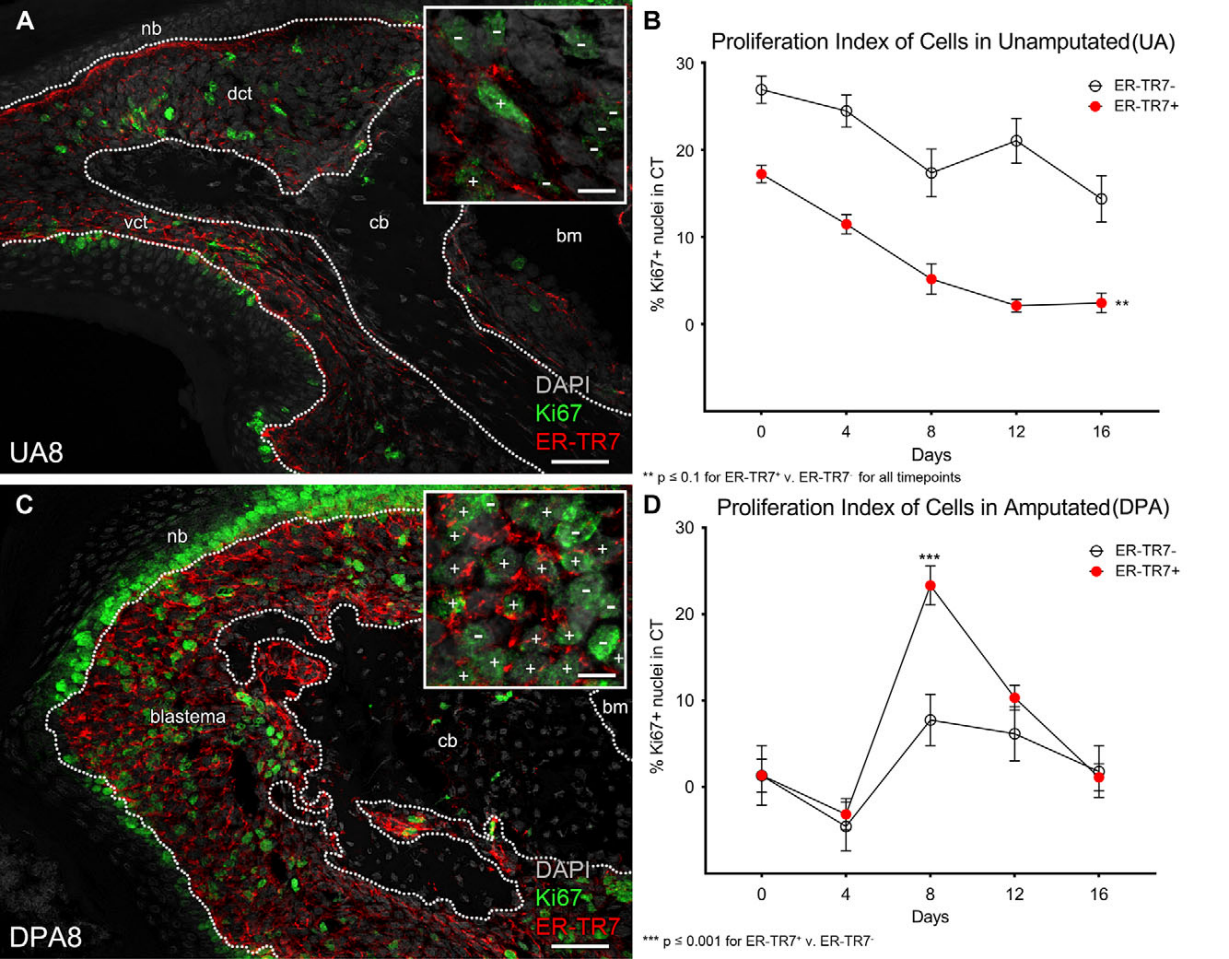
**Expansion of ER‐TR7^+^ FRCs is specific to the blastema stage**. A total of 500 cells were counted from random fields subsampled from the dorsal and ventral CT (dct and vct) or the blastema of neonatal controls and regenerates, respectively. The P3 bone marrow (bm) was excluded. In representative (A) UA8 and (C) DPA8 samples, these areas are flanked by the nail bed (nb) and the P3 bone (b) and outlined with a dotted white line (scale bar 50 μm). Proliferating (Ki67^+^) cells were grouped by ER‐TR7^+^ or ER‐TR7^−^ expression. (A), (C), insets: ER‐TR7^+^/Ki67^+^ cells were discriminated at 1000× magnification and are labeled with white + signs. ER‐TR7^−^/Ki67^+^ cells are marked with a − sign (scale bar 10 μm). (B) Measurement of the ratio of proliferating ER‐TR7^−^ cells to ER‐TR7^+^ cells in UA controls. (D) DPA8 blastema proliferating cells relative to ER‐TR7 reactivity. Data are presented as the mean ± SE (*n* = 4 per group)

The Ki67 proliferation index of ER‐TR7^+^ and ER‐TR7^−^ cell populations of control and regenerating digit tips was analyzed by co‐IHC staining (Fig. 4A−D). In UA controls, the proliferation index of both ER‐TR7^+^ and ER‐TR7^−^ cells displayed a steady decline over the course of this study. At all timepoints, the proliferation index of the ER‐TR7^−^ cells was significantly higher than that of the ER‐TR7^+^ subpopulation (*P* < 0.01, Fig. 4B). During the regeneration response, both cell populations display an initial drop in proliferation index at DPA4 and both display an increase in proliferation at the DPA8 blastema stage before returning to control levels at DPA16 (Fig. 4D). What is striking is that the relative increase in proliferating ER‐TR7^+^ blastema cells is much greater than for ER‐TR7^−^ blastema cells (Fig. 4D), indicating that the blastema microenvironment is selectively mitogenic for digit FRCs.

### 2.4 ER‐TR7^+^ FRCs represent a subpopulation of digit fibroblasts

**Figure 5:**
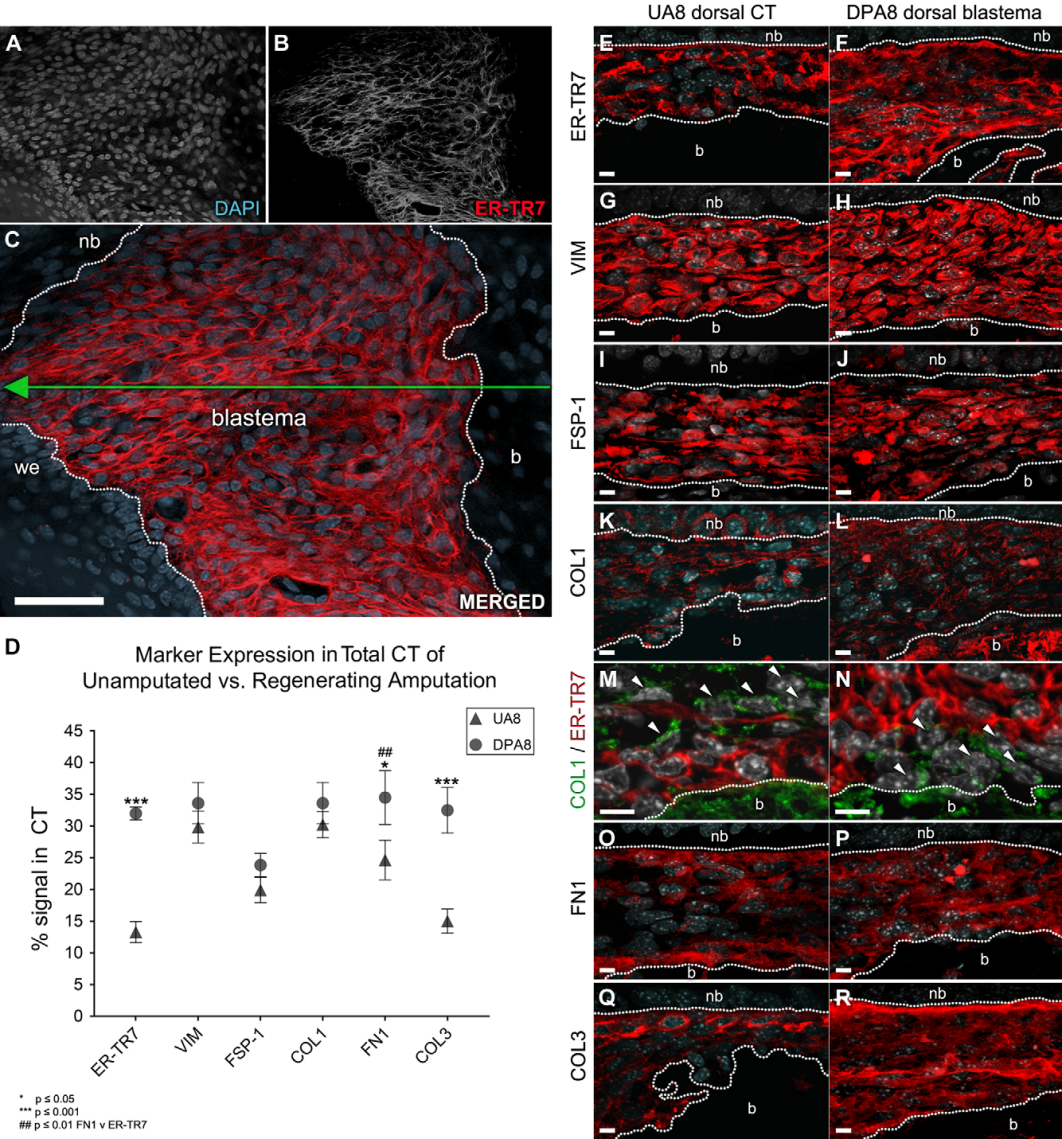
**Expression survey using fibroblast markers**. (A)−(C) Representative blastema (from *n* = 10 DPA8 digits) projection deconstructed to show grayscale channels of (A) blastema cell mass nuclei, (B) ER‐TR7^+^ organization, and (C) merged channel with surrounding compartments outlined (nb, nailbed; b, bone; we, wound epithelium; scale bar 50 μm). (D) Various antibodies against macromolecules and cytoskeletal proteins relevant to injury response from fibroblasts were detected by IHC, measured, compared as a percentage of individual marker staining over total CT area and plotted as mean ± SEM (*n* = 4 per group). (E)−(L), (O)−(R) Sections were immuno‐labeled in red for each marker and counterstained with DAPI (gray) on matched dorsal regions (boundaries outlined by white dotted lines between the nb and b) of UA8 or DPA8 sections and imaged at 600× magnification (scale bar 10 μm). (M), (N) Serial sections were also co‐stained against ER‐TR7 (red) and COL1 (green) to screen for non FRCs, that is, COL1^+^ /ER‐TR7^−^ fibroblasts (white arrows)

ER‐TR7 IHC identifies FRCs and an extracellular framework of fibers produced by them in the digit CT and blastema of regenerates. This ER‐TR7^+^ framework is organized in a honeycomb pattern that appears to orient along the proximodistal digit axis (Fig. 5A−C). The regeneration pattern appears distinct from the network present in non‐regenerating amputation wounds and an analysis of fiber organization confirms that, in contrast to the regenerating blastema, the ER‐TR7^+^ fibers in non‐regenerating wounds are arranged perpendicular rather than parallel to the proximodistal axis of the digit (Fig. S1A−F). Thus the orientation of the ER‐TR7^+^ matrix correlates with the polarity of the regenerative response.

ER‐TR7 IHC studies, especially in the discretely labeled UA adult digit tip relative to the amount of digit tip CT cells, suggest that digit FRCs represent a subpopulation of CT fibroblasts within the digit that are involved in blastema formation. However, fibroblasts in general remain a poorly characterized cell type and reliable cell‐type‐specific markers are not available. To begin to characterize fibroblasts associated with digit tip regeneration, IHC studies using a number of known fibroblast and CT markers were carried out. With the exception of anti‐COL1, antibodies of this fibroblast panel were detected by IHC individually in adjacent tissue sections of UA8 controls and DPA8 blastemas to screen for qualitative trends for expression similar to ER‐TR7 (Fig. 5E−P). In addition, a quantitative analysis of these fibroblast markers was performed comparing labeled regions of the dorsal CT of UA controls and in the blastema of regenerates to screen for differences in concentration, localization, and pattern to that observed in matched sections stained against ER‐TR7 (Fig. 5D). The IHC panel surveyed vimentin (VIM), a general mesenchyme marker that is ubiquitously expressed, fibroblast specific protein 1 (FSP‐1), COL1, COL3, and fibronectin (FN1).

VIM localizes to the cytoskeleton of all digit cells within the UA8 CT (Fig. 5G) and blastema mesenchyme (Fig. 5H). It serves as a control for ubiquitous expression, which does not change during blastema formation compared to the VIM^+^ CT of UA controls (Fig. 5D). FSP‐1, also known as S100A4, belongs to the S100 superfamily of calcium‐binding proteins (Strutz et al. 1995) and is reported to be specific to fibroblasts in organs undergoing remodeling (Lawson et al., 2005; Schneider et al., 2007; Zhang, Chen, Xiao, Wang, & Qin, 2011). Expression of FSP‐1 in the digit is localized to the cytosol of many CT and blastema cells (Fig. 5I and J). FSP‐1 expression is higher and localized to more cells in the UA8 CT compared to the ER‐TR7 labeled UA8 CT in an adjacent section (Fig. 5D, E, and I) and the difference between FSP‐1^+^ UA8 CT and FSP‐1^+^ DPA8 blastema (Fig. 5D, I, and J) tested insignificant (*P* > 0.05). We therefore conclude that changes in the pattern and level of FSP‐1 expression during blastema formation compared to UA controls are distinct from ER‐TR7.

COL1 is a fibril‐forming collagen synthesized and secreted by fibroblasts throughout the body. In UA8 digits, anti‐COL1 localizes to the cytosol of fibroblasts and to extracellular fibrils throughout the CT (Fig. 5K). In the blastema COL1 IHC identifies a dispersed network of fibers and cells that appear distinct from ER‐TR7 staining (Fig. 5L). COL1 is highly expressed in both the UA8 digit CT as well as the DPA8 blastema so its expression profile is uniquely different compared to matched ER‐TR7 samples. A detailed co‐IHC analysis of ER‐TR7 and COL1 expression identifies cells, based on cytosolic expression, in both the UA8 CT and the DPA8 blastema that are COL1^+^/ER‐TR7^−^ and COL1^−^/ER‐TR7^+^ (Fig. 5M and N). These observations provide evidence of two distinct fibroblast populations present in the digit CT and digit blastema.

FN1 is a glycoprotein secreted by fibroblasts which regulates composition of the CT matrix (McDonald, Kelley, & Broekelmann, 1982; Sottile et al., 2007; Velling, Risteli, Wennerberg, Mosher, & Johansson, 2002). FN1 staining in UA8 samples is diffuse but prominent below the stratum basale of the epidermis and associated with the periosteum (Fig. 5O). Between these areas of extracellular FN1 staining, isolated FN1^+^ cells around the vascular adventitia can be detected. Overall, these areas of FN1 expression roughly overlap areas where ER‐TR7^+^ cells are found. Following amputation, FN1 expression is homogeneous throughout the DPA8 blastema (Fig. 5P) and, like ER‐TR7, is upregulated over UA8 controls (*P* < 0.05; Fig. 5D). FN1 staining does not localize to fibrillar structures, but the FN1 expression pattern in the basal layers of the UA control digits and its increase during blastema formation are suggestive that digit FRCs are capable of producing FN1 or depend on its function.

COL3 is a primary component of reticular fibers and reticulin (Montes et al., 1980) of the ECM. COL3 is known to be expressed in the blastema (Simkin et al., 2015) and, when quantitated, displayed a regeneration expression profile that was similar to ER‐TR7 (*P* = 0.0002; Fig. 5D). Moreover, the general arrangement of COL3^+^ fibers in both DPA8 and UA8 groups, confirmed by fluorescence IHC (Fig. 5Q and R) and a modified Gridley silver stain for reticulin (Gridley, 1951), resembles the ER‐TR7^+^ expression pattern (Fig. S2).

**Figure 6:**
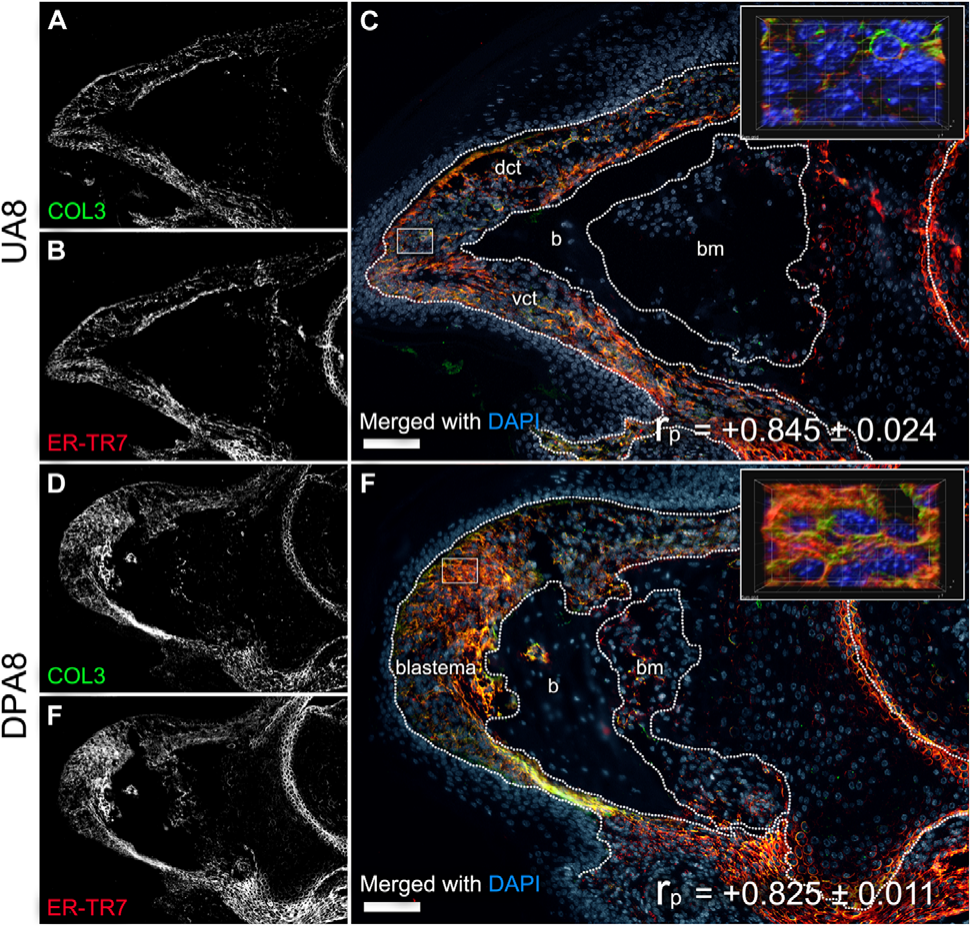
**ER‐TR7 shares an expression trend with COL3**. Representative (A)−(C) UA8 and (D)−(F) DPA8 samples from groups co‐stained for ER‐TR7 and COL3. Shown are individual (A), (D) COL3 and (B), (E) ER‐TR7 channels in grayscale with (C), (F) merged with DAPI color images (scale bar 100 μm). (C), (F), inserts: *Z*‐stacks captured from regions of interest at 600× magnification (white‐outlined rectangles on corresponding low magnification image) in UA8 and DPA8 samples were rendered in 3D format to depict the subcellular distribution of the markers (grid 10 μm). The trend between the two antigens in vivo was measured by PCC‐based analysis with mean ± SE values (*n* = 4) shown on corresponding (C), (F) merged channel images, where +1.0 is equivalent to a perfect trend and −1.0 equivalent to an absolutely opposite trend

To study a relationship between the ER‐TR7 and COL3, sections of DPA8 and UA8 digits (*n* = 5 per group) were co‐stained against the antigens, captured by confocal microscopy, and the resulting images were processed with a non‐bias co‐localization Pearson's correlation coefficient (PCC or *r*_p_) test. The PCC is reported on a scale of *r*_p_ values from 1 (perfect correlation) to −1 (perfect but negative correlation), where 0 equals no relationship. All sample sets yielded a strong correlation between both antigens, with mean *r*_p_ values of 0.845 ± 0.024 and 0.825 ± 0.011 for UA8 (Fig. 6A−C) and DPA8 (Fig. 6D−F) groups of digits, respectively. These data provide evidence of a link between ER‐TR7 and COL3 and a trend in their expression and pattern in and around digit FRCs in both the UA CT and regeneration blastema.

The results from this survey provide evidence of distinct fibroblast subpopulations in the digit CT and blastema. Moreover, the data identify FN1 and COL3 as fibroblast related proteins that display regulation profiles similar to ER‐TR7 in the digit tip and during blastema formation, which suggests they have a role in the dynamics of digit FRCs and regeneration in the blastema microenvironment.

### 2.5 ER‐TR7 can be induced in digit fibroblasts

**Figure 7:**
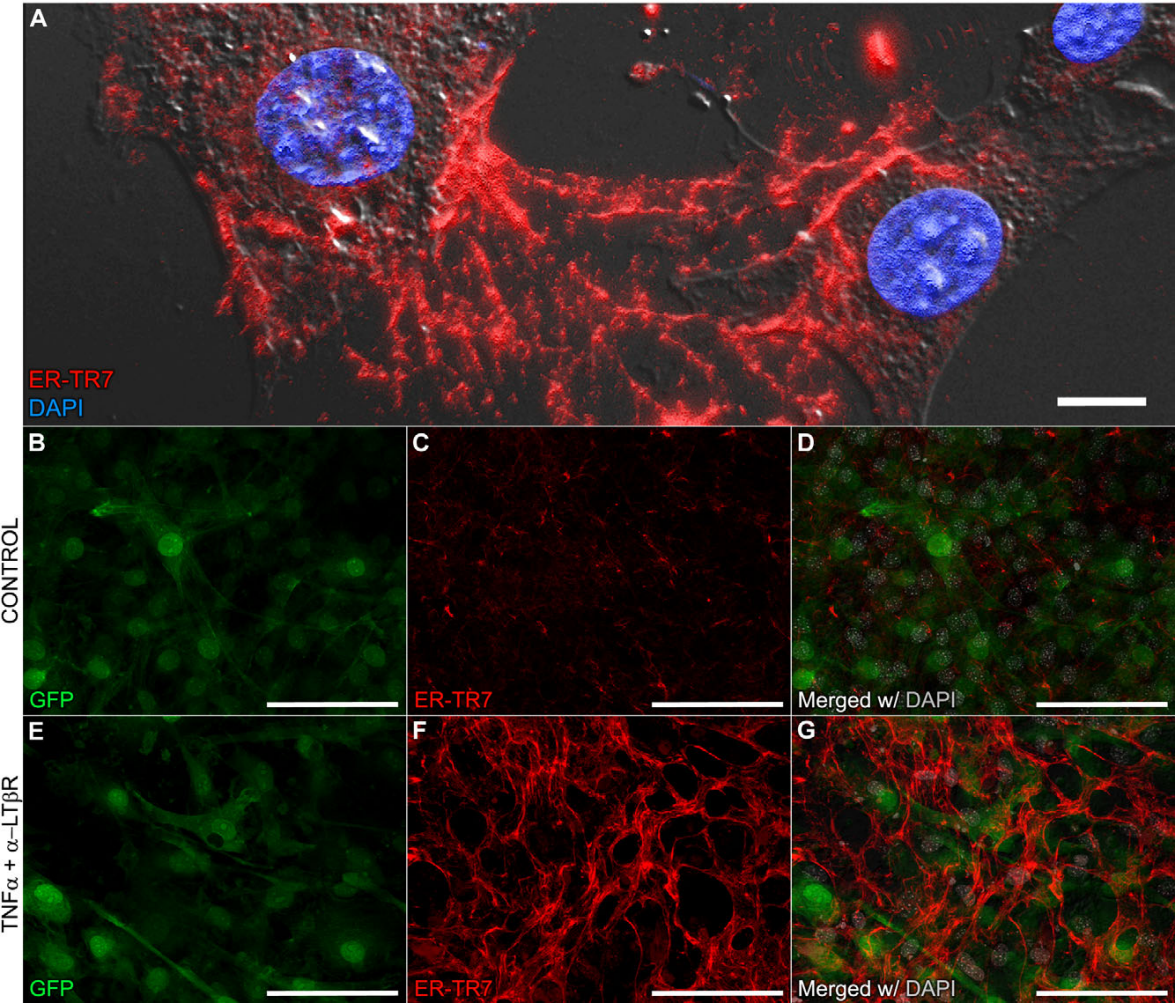
**ER‐TR7^+^ filament induction in P3 cells**. (A) Freshly isolated blastema cells retain the intercellular ER‐TR7 network in vitro (630×; scale bar 10 μm). (B)−(G) Cells isolated from digit tips harvested from eGFP transgenic mice were split into (B)−(D) untreated control and (E)−(G) TNFα + anti‐LTβR induced lines (representative fields at 400×; scale bar 50 μm)

Cultured cells derived from neonatal DPA8 blastemas (Lee et al., 2013) retain membranous and extracellular ER‐TR7^+^ fibril formation during expansion (Fig. 7A). The shape of cultured blastema cells ranged from spindle to stellate and, following ER‐TR7 immunocytochemistry (ICC), it was apparent that many of the cells were interconnected by ER‐TR7^+^ filaments. In contrast, P3 fibroblasts isolated from the digit tip (Wu et al., 2013) displayed a spindle phenotype and a low level of ER‐TR7 staining (Fig. 7B−D). Treating P3 cells with TNFα in combination with an agonistic antibody to LTβR stimulates ER‐TR7^+^ staining similar to lymphoid FRCs (Katakai et al., 2004b). Following induction, many of the P3 cells display a stellate morphology and produce a robust ER‐TR7^+^ network of fibrils (Fig. 7E−G). At the endpoint of treatment (11 days), the induced P3 culture was less cellular than controls and the ER‐TR7 staining pattern was well defined and uniform. These studies show that, in parallel with lymphoid stromal cells (Katakai et al., 2004b), digit derived fibroblasts can be induced to produce a robust ER‐TR7^+^ network of fibrils. These data are consistent with the conclusion that ER‐TR7 negative fibroblasts can be induced to participate in the production of the enhanced ER‐TR7 network associated with blastema formation.

Using the induction of P3 cells to produce the ER‐TR7^+^ network, we employed a Real‐Time polymerase chain reaction (PCR) array to analyze expression of 84 key genes associated with mouse fibrosis in control and induced P3 cells (Katakai et al., 2004b). P3 fibroblasts at days 4, 7, and 10 following induction with TNFα and anti‐LTβR were analyzed using the manufacturer's recommended restrictions for analysis and a greater than 2‐fold change in expression. We identified four genes that were downregulated at all timepoints—*Acta2* (smooth muscle actin), *Cav1* (caveolin 1), *Cxcr4* (chemokine [C−X−C motif] receptor 4) and *Thbs1* (thrombospondin 1)—and three genes that were upregulated at all timepoints—*NFkb1*, *Col3a1* (collagen type III) and *Dcn* (decorin). Quantitative data for transcripts with greater than 2‐fold expression at any given timepoint are provided in the Supporting Information (Table S1). Upregulation of *NFkb1* was predicted since recombinant TNFα coupled with anti‐LTβR stimulation of lymphoid FRCs induces ER‐TR7 in a nuclear factor κB (NFκB) pathway dependent manner (Katakai et al., 2004b). Of the remaining upregulated genes, decorin is a matrix proteoglycan associated with COL1 and *Col3a1* encodes for the target antigen detected by anti‐COL3 IHC in digit sections.

To determine whether there is a relationship between ER‐TR7 and COL3 in register with our in vivo data (Fig. 6), we carried out co‐IHC localization studies on P3 cells co‐treated with TNFα and anti‐LTβR. Co‐localization analysis based on the PCC approach over eight high resolution photomicrographs at 400× magnification from each culture was performed (Fig. S3). Trends in localization and intensity level for these two antigens in both uninduced (Fig. S3A−C) and treated (Fig. S3D−F) P3 cells were almost identical gauged by our qualitative observations and strong pixel correlation measurements with overall mean *r*_p_ values above 0.8. The qRT‐PCR results complemented by the ICC co‐localization data further support the conclusion that the ER‐TR7 antigen is linked either directly or indirectly to the expression of COL3 by digit FRCs.

## 3 DISCUSSION

In this study, the ECM in the mouse digit tip blastema is characterized by focusing on the antigen ER‐TR7 which identifies a population of FRCs involved in compartmentalizing lymph organs during development (Balogh et al., 2008; Katakai, 2012; Katakai et al., 2004b; Link et al., 2007; Van Vliet et al., 1986). The CT of the digit tip is similarly compartmentalized by ER‐TR7^+^ fibers which are localized to the periosteal boundary of the P3 phalanx, the basal boundary of the nail epidermis, and outlining the vasculature. Compared to IHC staining with other fibroblast markers, the evidence supports the conclusion that ER‐TR7^+^ cells identify a subpopulation of loose CT fibroblasts that delineate the boundaries between different tissue types of the digit tip. Those tissue interfaces are bone/CT, epidermis/CT, and vasculature/CT. This expression pattern is suggestive of the digit FRCs playing a role in establishing and/or maintaining the spatial organization of tissue types by producing a network of ECM fibers. The evidence indicates that COL3 is a primary component of this network, and that the ER‐TR7 antigen is closely associated with the network. ER‐TR7 staining and COL3 production are co‐induced in cultured P3 digit fibroblast cells following treatment with TNFα and an agonist for LTβR. In lymphoid stromal cells, the induction of ER‐TR7 by a similar treatment is dependent on activation of the NFκβ signal transduction pathway (Katakai et al., 2004b); thus the available evidence suggests that this signaling pathway in digit FRCs is instrumental for the production of this ECM network.

During neonatal and adult digit tip regeneration, ER‐TR7 is upregulated as the blastema forms and is downregulated as the blastema differentiates. The changes in ER‐TR7 expression and its pattern were not observed in analogous CT areas of the digit expressing COL1 or FSP‐1. The blastema is composed of a large population of ER‐TR7^+^ digit FRCs and these cells co‐express COL3 but not COL1. The cellular staining of digit FRCs by means of the ER‐TR7 antibody in the blastema coupled with the ER‐TR7^+^ fibers which originate from these FRCs define the blastema ECM network and provide evidence that the distinctive provisional matrix of the blastema is produced by digit FRCs. We do not observe obvious compartmentalization within the blastema by ER‐TR7^+^ fibers. Instead, the network appears ubiquitous but loose and organized along the proximodistal axis of the blastema. This is in contrast to the ER‐TR7^+^ fibers associated with the non‐regenerating amputation wound that are tightly arranged perpendicular to the bone stump and similar to the fibrous cap which forms following conventional wound healing (Dawson et al., 2016; Turner, Johnson, & Badylak, 2010). While the blastema is predicted to be largely composed of heterogeneous populations of lineage restricted progenitor cells (Lehoczky et al., 2011; Rinkevich et al., 2011), the blastema itself lacks any overt organization that delineates cells derived from different tissues of the amputated digit tip. While it is possible that ER‐TR7^+^ cells in the amputated digit tip have the capability of proliferating and participating as FRCs in the blastema, testing this hypothesis will require a lineage marker which is unavailable at this point in time. Nevertheless, a link between the amputated digit tip and blastema FRCs is supported by the significant decline in ER‐TR7 staining associated with the localized degradation of the amputated bone that is characteristic of adult digit tip regeneration (Fernando et al., 2011) and the enhanced proliferation of FRCs within the blastema. The data suggest that the enhanced level of ER‐TR7 staining associated with the blastema represents a combination of a relative increase in FRC numbers coupled with an increase in ER‐TR7^+^ matrix production. As regeneration proceeds, the overall level of ER‐TR7 staining returns to pre‐amputation levels indicating that the ER‐TR7^+^ fiber network is degraded or otherwise remodeled as the blastema differentiates. These data indicate that the blastema consists of a provisional ECM network produced by digit FRCs that is re‐modeled as the newly regenerated tissues mature. We propose that this provisional blastema network plays a critical role in recruitment and organization of progenitor cells during a regenerative response and, in this regard, the character of this provisional matrix can instruct the design of engineered biological matrices necessary for successful therapies in regenerative medicine.

In vitro studies involving lymphoid FRCs have demonstrated that the ER‐TR7^+^ network of fibers can be induced by treatment with TNFα and an LTβR receptor agonist antibody (Katakai et al., 2004b). These ligands and their receptors, both belonging to the TNF superfamily, have been known to trigger a milieu of pro‐inflammatory factors during injury and host defense in an NFκB‐dependent manner (Hehlgans & Pfeffer, 2005; Hehlgans, Muller, Stopfer, & Mannel, 2003; Katakai et al., 2004b) as well as stimulating homeostasis during lymphoid organogenesis (Daller et al., 2011; Hehlgans et al., 2002; Kahaleh, Smith, Soma, & LeRoy, 1988; White et al., 2007; Zeng et al., 2012). A similar treatment of fibroblasts derived from the mouse digit tip also triggers ER‐TR7^+^ fibril production that forms a fibril network similar to cultured blastema cells. The multi‐TNF receptor activation of fibroblast subtypes elicits progression of the canonical (RelA [p65]−p50 complex) and alternative (RelB−p52 [p100] complex) NFκB cascades (Katakai et al., 2004b), and can have a profound effect in a wide variety of physiological processes (Ghosh & Hayden, 2008). In fibroblasts, the pleiotropic nature of TNF is portrayed by in vitro studies which demonstrate opposite phenotypes on the synthesis and regulation of ECM macromolecules following activation of TNF receptor(s) suggesting that these events vary in a cell‐ or tissue‐specific manner (Distler, Schett, Gay, & Distler, 2008). The demonstration that ER‐TR7^+^ fibers are induced in cultured digit fibroblasts by multi‐TNF receptor activation coupled with the evidence showing that ER‐TR7^+^ fibers are induced in vivo during blastema formation provide evidence implicating TNF signaling as critical for the production of the blastema ECM network.

IHC co‐staining for ER‐TR7 and COL3 displays a high Pearson's coefficient indicating that the ER‐TR7 antigen is tightly linked to COL3 within the regenerating blastema. During the healing of full‐thickness skin wounds, the granulation tissue that forms has a high COL3 content and is thought to promote cell migration during wound healing (Barnes, Morton, Bennett, Bailey, & Sims, 1976). COL3 levels are reported to be higher in fetal wounds that are able to heal without scarring compared to scar‐forming adult wounds (Leung, Crombleholme, & Keswani, 2012). In animals that can undergo a scar‐free healing response, such as the spiny mouse (*Acomys*) or the FoxN1 deficient (nude) mouse, studies show that COL3 upregulation correlates with a regenerative response (Brant, Yoon, Polvadore, Barbazuk, & Maden, 2016; Gawronska‐Kozak & Kirk‐Ballard, 2013; Seifert et al., 2012). On the other hand, reducing COL3 in granulation tissue promotes the differentiation of myofibroblasts, enhances wound contraction, and increases the deposition of scar tissue (Volk, Wang, Mauldin, Liechty, & Adams, 2011). Thus, the demonstration that COL3 fibers are a prominent component of the blastema scaffold adds to the circumstantial evidence from multiple regenerative models that supports the conclusion that a COL3‐based provisional matrix provides a scaffold that promotes successful regeneration. It seems likely that the ER‐TR7 antigen represents a modification of this regenerative scaffold; however, its role in regeneration will have to await the identification of the antigen.

In a previous study, we characterized fibroblasts derived from the regeneration‐competent P3 digit region and compared them to fibroblasts derived from the regeneration‐incompetent P2 digit region (Wu et al., 2013). While both P2 and P3 cells are able to participate in blastema formation, the regeneration‐competent P3 cells displayed position‐specific characteristics in their interaction with epidermal cells in vitro and in the way they interacted with the ECM when cultured under different conditions (Wu et al., 2013). In humans, fibroblast cells have been singled out as the primary cell type that maintains distinct patterns of gene expression that vary with spatial position across the body; thus they are poised to play an essential role in conveying spatial information to cells important for a successful regenerative response (Chang et al., 2002; Rinn, Bondre, Gladstone, Brown, & Chang, 2006). In amphibian limb regeneration, there is considerable indirect evidence that fibroblasts of the dermis play an early role in forming the blastema and in relaying positional information that organizes the pattern of regenerating structures (Bryant, Gardiner, & Muneoka, 1987; Bryant et al., 2002; Nacu et al., 2013). The ability of P3 fibroblasts to respond to signals derived from the immune response by producing an ER‐TR7^+^ ECM network adds to the evidence linking fibroblasts to the control of regeneration, and suggests a specific function in producing a provisional matrix template that dictates the structure of the regenerate. These data are also consistent with studies in other regenerating models, including liver regeneration, that implicate signaling by the immune system as essential for a successful regenerative response (Sorg et al., 2016; Tumanov et al., 2009). We also note that, during regeneration, the digit FRCs proliferate and create a provisional blastema matrix that becomes populated by other cell types (i.e., ER‐TR7^−^ cells) in much the same way that lymphoid FRCs undergo hypertrophy to create the microenvironment necessary around ER‐TR7^−^ components of blood vessels, lymphatics, and lymphocyte compartments for a successful adaptive immune response in the lymph node (Chyou et al., 2011). Overall, fibroblasts play a role in positional recognition and growth regulation that is critical for a regenerative response. This is surprisingly similar to the role that, in addition to cell growth and migration, lymphoid FRCs play in regulating antigen recognition and presentation in an adaptive immune response, and suggests an evolutionary relationship between these two complex responses to homeostatic disruption.

## 4 MATERIALS AND METHODS

### 4.1 Mice and tissue harvest

All neonate and adult subjects consisted of outbred CD1 mice supplied by Charles River Laboratories (Wilmington, MA, USA). PN3 neonates and 8W adults were anesthetized with an intraperitoneal injection of ketamine and xylazine at 80 and 8 mg/kg of body weight, respectively, followed by distal amputation of digits 2 and 4 from each hind limb using microdissection scissors (neonate) or a scalpel (adult) under a stereomicroscope, as previously described (Fernando et al., 2011; Han et al., 2008). For preliminary and supporting data purposes, a number of adult mice underwent amputations midway through P2 of digits 2 and 4 with a scalpel, as previously described (Dawson et al., 2016). Digit tissues were harvested for histological and IHC analysis at DPA0, 4, 8, 12, and 16 for neonates and DPA0, 5, 10, 14, 21, 28, and 35 for adults. Procedures for care and use of mice for this study were performed in accordance with standard operating procedures approved by the Institutional Animal Care and Use Committee of Tulane University and Louisiana State University Health Sciences Center in New Orleans, LA.

### 4.2 Histology and fluorescence IHC

Tissues were harvested in zinc‐buffered formalin (Anatech Ltd, Battle Creek, MI, USA) to fix no longer than 48 h and decalcified in formic‐acid‐based solution for 15−18 h (Decal I; Surgipath, Richmond, IL, USA) for paraffin processing and thin section histopathology and IHC. Serial tissue sections were collected in a rotary microtome at 5 μm, floated on a water bath at 40°C, mounted onto charged, glass slides and heated for 45 min at 60°C in an oven. One set of slides was chemically treated to stain for reticular fibers by the modified Gridley reticulin method (Gridley, 1951) or H&E staining. The remaining slides were deparaffinized in xylene and rehydrated through an ethanol series to distilled water. All washes were performed with phosphate‐buffered saline (PBS). Depending on the antigens to be detected, sections were pre‐treated with either incubation with an enzyme (Proteinase K, Dakocytomation, Carpinteria, CA, USA) at 37°C for 10 min or heat induced epitope retrieval (target retrieval buffer, pH 9.0, Dakocytomation) in a pressure cooker followed by cooling for 20 min and washed in PBS. This was followed by blocking for nonspecific protein binding with 5% goat serum diluted in 1% (w/v) bovine serum albumin (BSA) for 15 min at room temperature. It should be noted that, if using a primary antibody derived from mouse for subsequent tagging with an anti‐mouse secondary antibody, an additional goat anti‐mouse Ig Fab fragment (Jackson Immunoresearch, Westgrove, PA, USA) was added to the protein block step at 10 μg/mL for 1 h at room temperature to bind endogenous immunoglobulins. Following washing, sections were single or co‐incubated overnight at 4°C with a variety of primary antibodies: rat anti‐ER‐TR7 (clone ER‐TR7; 5 μg/mL; catalog # MCA2402; Abd Serotec, Raleigh, NC, USA), mouse anti‐smooth muscle actin (SMA; clone 1A4; 2 μg/mL; catalog # M0851; Dakocytomation); rabbit anti‐von Willebrand factor (FVIII; polyclonal; 1 μg/mL; catalog # A0082; Dakocytomation); rabbit anti‐osteocalcin (OC; polyclonal; 1 μg/mL; catalog # MK127; Takara, Otsu, Shiga, Japan); rat anti‐Ki67 (clone TEC‐3; catalog # M7249; 5 μg/mL; Dakocytomation); rabbit anti‐cleaved caspase 3 (C3; polyclonal; 5 μg/mL; catalog # 9661; Cell Signaling, Danvers, MA, USA); rabbit anti‐VIM (clone SP20; 1 μg/mL; catalog # RM‐9120‐S1; Labvision, Fremont, CA, USA); rabbit anti‐COL1 (polyclonal; 1 μg/mL; catalog # NB600‐408; Novus Biologicals, Littleton, CO, USA); rabbit anti‐COL3 (polyclonal; 2 μg/mL; catalog # ab7778; Abcam, Cambridge, MA, USA); rabbit anti‐S100A4 (FSP1; polyclonal; catalog # ab27957; 1 μg/mL; Abcam); rabbit anti‐fibronectin (FN1; polyclonal; 2 μg/mL; catalog # ab2413; Abcam); and syrian hamster anti‐gp38 (clone RTD4E10; 5 μg/mL; catalog # ab11936; Abcam). Labeling of bound primaries was followed by indirect IHC using Alexa‐conjugated goat F(ab′)2 secondary antibodies (Molecular Probes, Eugene, OR, USA) against the primaries’ respective host species at a concentration of 4 μg/mL diluted in 300 nM of DAPI (Molecular Probes) in PBS for 1 h at room temperature. Slides were washed in PBS and mounted under a coverslip with Prolong Gold Antifade (Molecular Probes) for epifluorescence deconvolution or confocal microscopy.

### 4.3 ER‐TR7 induction in P3 cells

Primary cells from the P3 digit region of adult 8W male CD1 (Charles River) or C57BL/6‐TGN(ACTB‐eGFP) transgenic (Jackson Laboratory, Bar Harbor, ME, USA) mice were collected to generate untagged or enhanced green fluorescent protein (eGFP) tagged cell lines, respectively, as previously described (Wu et al., 2013). These P3 cell lines were plated on fibronectin‐coated chamber slides (Corning, Corning, NY, USA) at a concentration of 1 × 10^5^ per chamber. Cells either remained untreated or were stimulated to produce the ER‐TR7^+^ network according to procedures previously described (Katakai et al., 2004b). Briefly, cells were allowed to adhere and recover from trypsinization for 24 h at which time they were co‐treated with 100 ng/mL of recombinant TNFα (catalog # 410‐MT; R&D Systems, Minneapolis, MN, USA) and anti‐LTβR (catalog # AF1008; R&D Systems) at a concentration of 1 μg/mL. This treatment was reapplied at days 3, 6, and 9. At day 11, untreated and induced cells were collected for qRT‐PCR or fixed for IHC.

### 4.4 RNA extraction and Real‐Time PCR

Total RNA was isolated from untreated control and experimental P3 cell lines using Trizol Reagent (Invitrogen, Carlsbad, CA, USA). Following DNase treatment, RNA was purified using the Qiagen RNeasy Mini Kit (Qiagen, Valencia, CA, USA) and its quality was determined using a Nanodrop 2000 (Thermo Fisher Scientific Inc., Waltham, MA, USA). cDNA was synthesized by RT^2^ First Strand Kit (SABiosciences, Frederick, MD, USA) following the manufacturer's instructions. Expression profile was assessed using a Mouse Fibrosis RT^2^ Profiler PCR Array and labeled with RT^2^ qPCR SYBR green PCR Master Mix (SABiosciences) according to the manufacturer's recommended protocols. Quantitative PCR was performed with a LightCycler 480 system (Roche Applied Sciences, Indianapolis, IN, USA) and its software was used to determine a critical threshold, which was the cycle number where the linear phase for each sample crossed the threshold level. Relative gene expression was determined using critical threshold methods. Data were further analyzed by SABiosciences PCR array data analysis online tools (http://pcrdataanalysis.sabiosciences.com/pcr/arrayanalysis.php).

### 4.5 Immunocytochemistry (ICC)

Cell preparations on chamber slides were washed in pre‐warmed PBS, fixed in pre‐warmed 4% methanol‐free formaldehyde (Polysciences, Warrington, PA, USA), and washed in PBS. Samples were permeabilized in acetone at −20°C, washed, and treated with 5% normal goat serum in 1% (w/v) BSA in PBS to reduce nonspecific binding. Subsequently, cells were co‐incubated for 3 h at room temperature with rat anti‐ER‐TR7 (5 μg/mL; Abd Serotec) and rabbit anti‐COL3 (2 μg/mL; Abcam) or rabbit anti‐COL1 (1 μg/mL; Novus Biologicals). Primary antibodies were detected by indirect immunofluorescence using a goat antibody against the primary antibodies’ source species conjugated to Alexa dyes at 4 μg/mL with added DAPI at 300 mM. Samples were mounted under coverglass with Prolong Gold Antifade.

#### 4.5.1 Microscopy

Tissue sections were imaged using a Leica DMRXA upright microscope equipped with a Sensicam QE CCD (Cooke Corporation, Romulus, MI, USA), *xyz* motorized stage (Prior Scientific, Rockland, MA, USA), an Hg source, and filters suitable for DAPI, Alexa 488, Alexa 594, and Alexa 647 fluorophores. Additional photomicrographs from tissue sections and ICC preparations were also captured with a Fluoview FV1000 laser scanning confocal system (Olympus of America, Center Valley, PA, USA) equipped with Nomarski (differential interference contrast or DIC) and visible excitation light sources including a multi‐line argon laser and diodes covering 405, 561, 592, and 635 nm wavelengths. False positive results arising from autofluorescence mostly inherent to areas of high keratin accumulation, elastic fibers, and porphyrins in erythrocytes (Croce & Bottiroli, 2014) were meticulously segregated by sequential co‐registration of true positive captures in parallel with channels in adjacent but unmixed spectral ranges. In addition, fluorescence of fully treated tissue sections was compared to that of corresponding serial sections in which the primary antibody was replaced by an isotype control antibody diluted to the same concentration.

No Neighbors and Constrained Iterative deconvolution algorithms were applied to 2D and 3D sets, respectively. Post‐imaging measurements included protein expression profiles based on detection areas (both in vitro and in vivo samples) and cell counts. Area measurements of the expression of ER‐TR7 and other markers were performed with Slidebook software by masking images with a binary layer encompassing specific minimum and maximum fluorophore detection intensities. The areas were automatically calculated by the software in pixel values and these in turn were divided over the total nuclear or tissue area, which was masked using DAPI intensities and/or anatomical landmarks. The anatomical landmarks confining the digit CT analyzed are the nailbed epithelium, the periosteum, the joint (proximally) and the tendon enthesis (ventrally). For cell number analyses, events were manually quantified with a cell counter and annotated over total nuclei per field. Co‐localization analyses using Pearson's correlation coefficients were calculated within thresholded areas of co‐stained antigens. All renderings and analyses were driven by Slidebook software (Intelligent Imaging Innovations, Denver, CO, USA). Supporting data analysis of fiber arrangements between adult P3 and P2 amputations was performed by generating and measuring vectors in 32‐bit thresholded channels of ER‐TR7^+^ staining using the OrientationJ plugin (Fonck et al., 2009; Rezakhaniha et al., 2012) available online at http://bigwww.epfl.ch/demo/orientation for the digital image processing software ImageJ.

### 4.6 Statistical analysis

In all cases, quantitative data are represented as means ± standard error of the mean (SEM). Prism (version 7.01, GraphPad Software, La Jolla, CA, USA) was used to perform statistical analyses. The significance between fibroblast marker stained areas of UA8 and DPA8 groups was determined via unpaired *t* tests with two‐tailed distributions. A one‐way ANOVA with Sidak corrected post hoc tests was applied to all line graphs. In all cases, a value of *P* < 0.05 was deemed statistically significant.

## Supporting information


*Fig. S1*.

*Fig. S2*.

*Fig. S3*.

*Table S1*.
