# Supplementary figures and images for: Fibroblast reticular cells engineer a blastema extracellular network during digit tip regeneration in mice
Source: Regeneration (Oxf). 2017 May 3;4(2):69–84. doi: 10.1002/reg2.75 (PMC5469731; doi:10.1002/reg2.75)

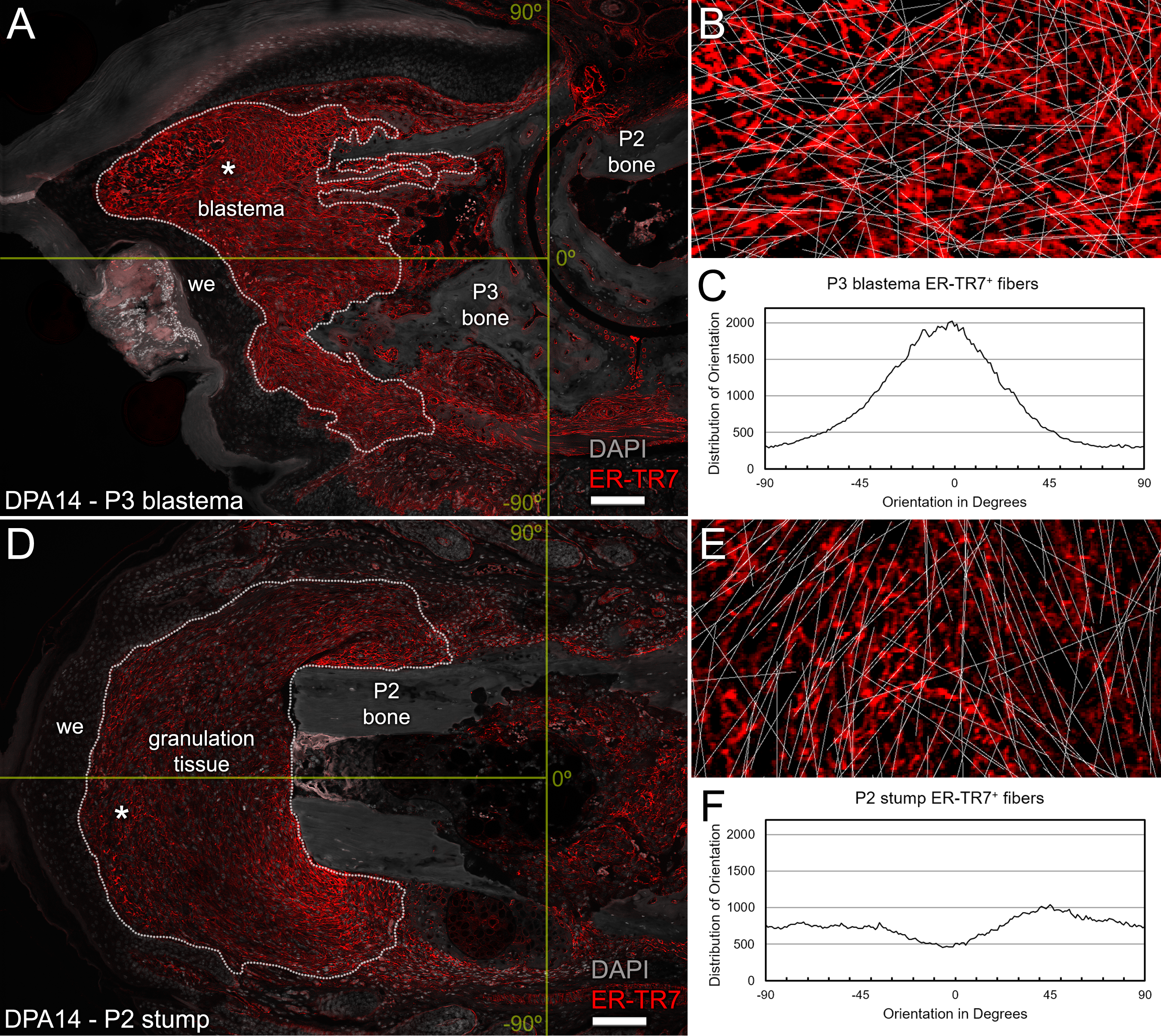

Supplement: Supplementary file 1 — Fig. S1. [file REG2-4-69-s001.tif]

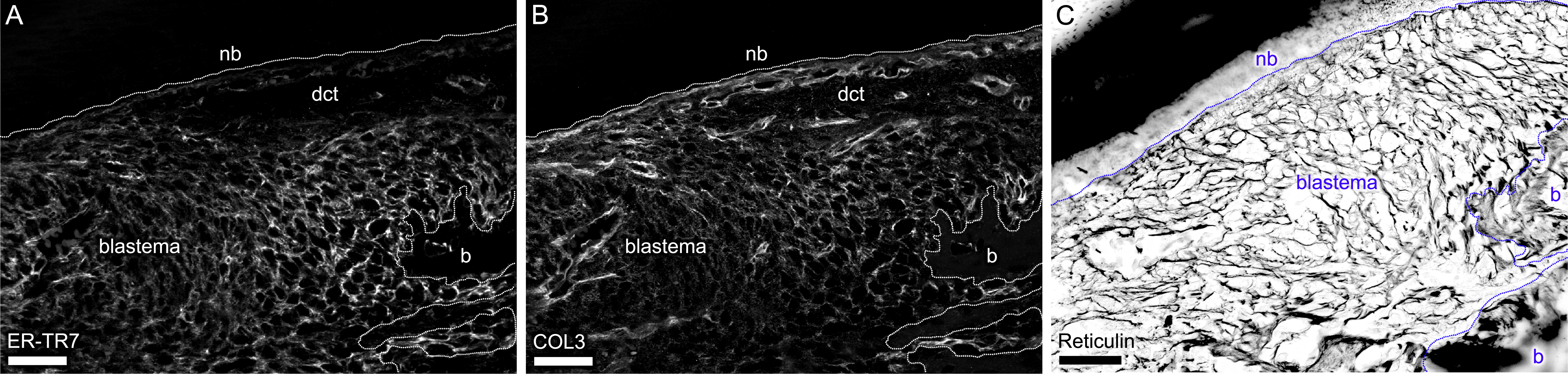

Supplement: Supplementary file 2 — Fig. S2. [file REG2-4-69-s002.tif]

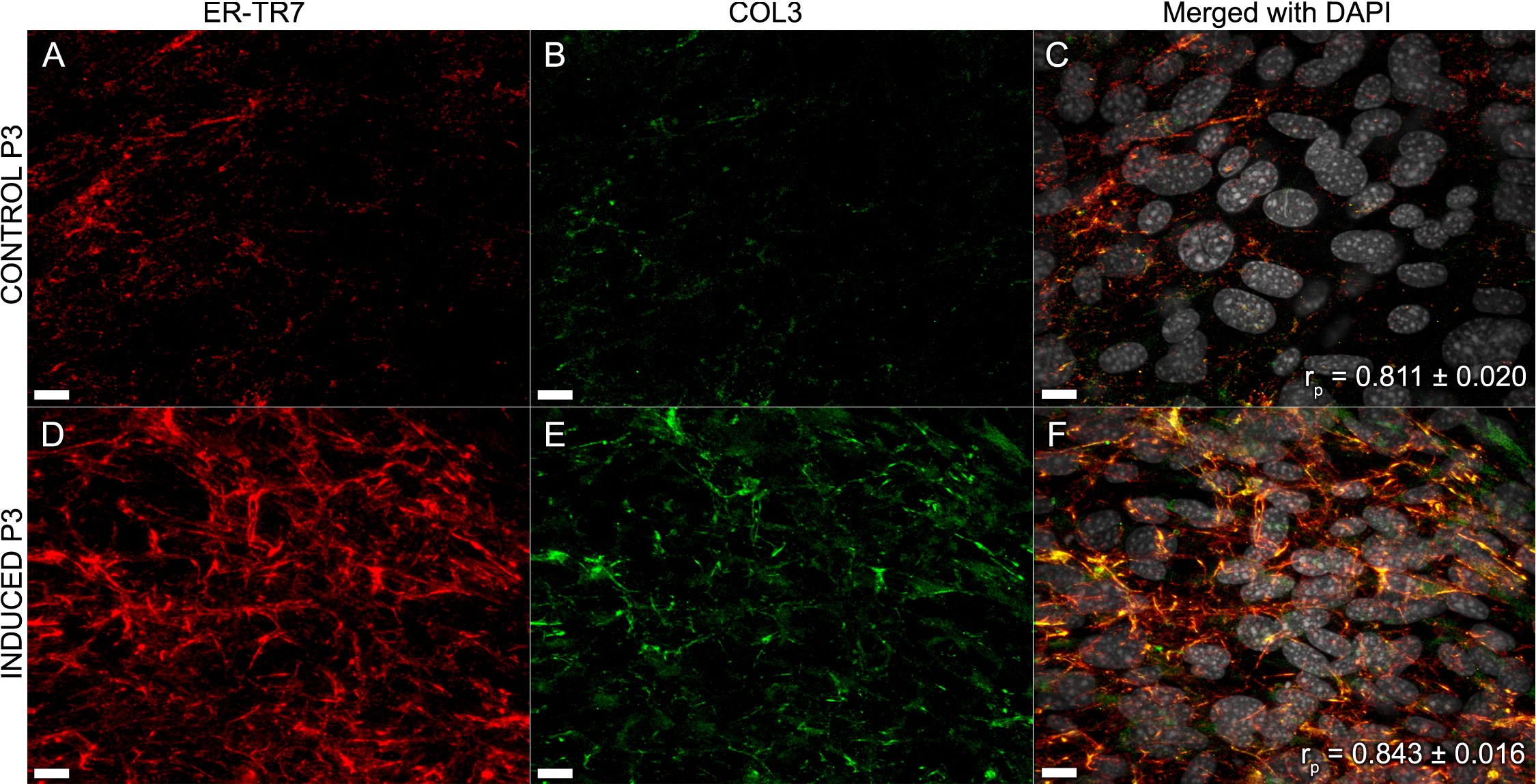

Supplement: Supplementary file 3 — Fig. S3. [file REG2-4-69-s003.tif]
