# Supplementary material for: Fibroblast reticular cells engineer a blastema extracellular network during digit tip regeneration in mice
Source: Regeneration (Oxf). 2017 May 3;4(2):69–84. doi: 10.1002/reg2.75 (PMC5469731; doi:10.1002/reg2.75)
Supplement: Supplementary file 4 — Table S1. [file REG2-4-69-s004.docx]

| **Gene Symbol** | **Gene Description** | **Fold Change** | | |
| --- | --- | --- | --- | --- |
|  |  | **Day 4** | **Day 7** | **Day 10** |
| *Acta2* | actin, alpha 2, smooth muscle, aorta | -7.16 | -2.74 | -3.93 |
| *Cav1* | caveolin 1, caveolae protein, 22kDa | -31.13 | -8.43 | -2.98 |
| *Col1a1* | collagen, type I, alpha 1 | -3.29 | -1.29 | -2.08 |
| *Col3a1* | collagen, type III, alpha 1 | 2.15 | 6.29 | 5.96 |
| *Ctgf* | connective tissue growth factor | 2.03 | -11.12 | -4.61 |
| *Cxcr4* | chemokine (C-X-C motif) receptor 4 | -10.13 | -2.86 | -2.80 |
| *Dcn* | decorin | 81.01 | 1957.78 | 7206.09 |
| *Il13ra2* | interleukin 13 receptor, alpha 2 | -3.41 | 1.26 | 16.62 |
| *Ilk* | integrin-linked kinase | -1.56 | -2.24 | -1.08 |
| *Itga2* | integrin, alpha 2 (CD49B, alpha 2 subunit of VLA-2 receptor) | 75.58 | 125.80 | 81.86 |
| *Nfkb1* | nuclear factor of kappa light polypeptide gene enhancer in B-cells 1 | 3.13 | 4.27 | 2.24 |
| *Smad7* | SMAD family member 7 | 3.30 | 1.68 | 3.49 |
| *Tgfbr1* | transforming growth factor, beta receptor 1 | -2.01 | -1.39 | 1.39 |
| *Tgfbr2* | transforming growth factor, beta receptor II (70/80kDa) | -2.20 | -1.63 | 1.29 |
| *Thbs1* | thrombospondin 1 | -7.46 | -2.63 | -3.22 |
| *Thbs2* | thrombospondin 2 | -17.88 | -4.30 | 1.11 |
| *Timp3* | TIMP metallopeptidase inhibitor 3 | -5.10 | 1.86 | -1.13 |
| *Gapdh* (control) | glyceraldehyde-3-phosphate dehydrogenase | -1.05 | -1.01 | -1.04 |
| *Actb* (control) | actin, beta | 1.65 | 1.61 | 1.04 |

Supporting Table S1. *ER-TR7 induction of P3 cells promotes upregulation of Col3a1 gene transcripts.* A mouse gene array consisting of 84 fibrosis-related transcripts was used to analyze modulation of gene expression in P3 cell line lysates following treatment with recombinant TNFα and anti-LTβR shortly after days 4, 7, and 10 of the ER-TR7 induction treatment period. Listed is the subset of genes that yielded greater than 2-fold change from uninduced control cell values in one or more timepoints.
